# Common computed tomography artifact: source and avoidance

**DOI:** 10.1186/s43055-021-00530-0

**Published:** 2021-06-18

**Authors:** Amel F. Alzain, Nagwan Elhussein, Ibtisam Abdallah Fadulelmulla, Amna Mohamed Ahmed, M. E. Elbashir, Badria Awad Elamin

**Affiliations:** 1 Radiological Science Department, College of Applied Medical Science, Taibah University, El-Madinah, El Monawara, Saudi Arabia; 2 Diagnostic Radiology Department, College of Applied Medical Science, University of Hail, Hail, Saudi Arabia; 3 Radiological Science Department, Alghad International College for Applied Medical Science, Tabuk, Saudi Arabia; 4 Radiological Science Department, College of Applied Medical Science, Taif University, Taif, Saudi Arabia

**Keywords:** Computed tomography, Artifacts, Image quality, Artifact source, Artifact reduction

## Abstract

**Background:**

Artifacts have significantly degraded the quality of computed tomography (CT) images, to the extent of making them unusable for diagnosis. The types of artifact that could be used are as follows: (a) streaking, which is commonly due to a discrepancy in a single measurement, (b) shading, which is due to a group of channels deviating gradually from the true measurement, (c) rings, which are due to errors in individual detector calibration and (d) distortion, which is due to helical reconstruction. It is occasionally possible to avoid scanning of a bony area, by means of changing the postion of the patient. Thus, this study aimed to evaluate the common artifacts that affect image quality and the method of correction to improve image quality.

**Results:**

The data were collected by distributing a questionnaire to the CT technologist at different hospitals about the most common type of artifacts in the CT images, source of artifacts and methods of correction. A total of 95 CT technologists responded to the questionnaire, which included 67% males and 33% females. Most of the participants (70%) were experienced CT technologists, and 61% of the participants had not done any subspecialty CT scan courses. The most common artifact used in the CT departments was motion artifact in brain CT (73%), and the best method to reduce motion artifact was patient preparation (87%).

**Conclusions:**

The most common shown artifact in this study was motion artifact, and the common cause was the patient-based artifact. It is important to understand why objects occur and how they could be prevented or suppressed to improve image quality.

## Background

Computed tomography (CT) examinations are extensively used in medical diagnosis and post-operative assessments. However, the artifacts are induced by a metal implant that might severely degrade picture quality and therefore, affect quality of diagnostic correctness. Particularly the existence of high-density substances, such as dental stuffing, hardening and photon starvation [1], therefore decreasing metal artifacts. However, these methods are extremely dependent on the arrangement of the metal material and might not be fully operative for transplants with high diminution constants like dental fillings and hip implant [2]. In certainty, rays that are passing to or adjacent metal implants are highly diminished and have a much greater fault because of an amalgamation of scatter, beam-hardening effects, noise from low photon count edge effects, and patient motion.

At the most initial stage, a CT image artifact is an inconsistency amongst the recreated values in a CT image and is based on the material thickness and the simple geometry. They are characteristically manifested as bright or dark bands or shades that typically follow some sort of forecast outline. There are two initial categories of artifacts: one produced due to difficulties with the CT imaging setup (e.g. ring artifacts, under-sampling aka aliasing, sample movement, etc.) and the other one which is more sample dependent such as beam hardening, scattered radiation, and lack of x-ray infiltration [3].

The quality of image and measurements include subjective evaluation of three skilled radiologists (R1, R2, R3), with at least 10 years of experience in general diagnostic radiology, and self-sufficient measurement of the image excellence using a 0–3 score scale [4]. Score 0 means that the image artifact is clear, the bony structure and soft tissue near the artifact cannot be observed and diagnosed, reasonable artifacts are seen in the image; the unpolished structure could still be detected, but impact the diagnosis, slight artifacts and adjacent anatomic constructions might be detected and diagnosed.

In computed tomography (CT), metal artifacts happen because of the occurrence of highly attenuating materials, that is, prostheses and dental fillings, in a scanning field of view. Naturally, severe streaking artifacts among dense objects are seen after image reconstruction. These artifacts obscure the adjacent structures, mainly the soft tissues, which could lead to improper tissue identification and description for diagnostics or wrong dose calculation for radiation therapy treatment planning [5]. Patient motion, which generates conflicts within the developed projection data, is a major cause of artifacts in clinical x-ray computed tomography (CT). A process of rewarding for head motion, which might be considered almost stiff, in helical CT would be of significant help when imaging children, as well as patients experiencing dementia or head trauma. Some reviewing motion modification techniques have been planned to recompense for head motion [6], including avoidance of metal artifacts by the operator, and built-in features for minimizing motion artifacts.

Another form of artifact is known as scanner-based artifiact, and it is comprised of ring artifacts, incomplete projection, avoidance and software corrections.

This study was conducted to assess the common artifacts that exist in CT scan imaging according to the examination done in the CT department, different types of artifact within each of these categories were described with regard to (a) the mechanisms of generation, (b) methods employed by the CT technologist to suppress them and (c) techniques for artifact avoidance available to the operator.

## Methods

In this study, a quantitative method was selected. The study surveyed around 95 participants. About 9 questions were organized and were encompassed in the questionnaire; the data were collected from survey. The study examined the outcomes based on demographic and evocative examination. It was remarkable that the members belonged to different areas, mindset, and circumstances. Therefore, the outcomes were based on the defendants’ discernment.

Questionnaire was dispersed among the members of different hospitals including the majority of BSC (79) and MSC (7) students and also the students of PhD (1) and diploma (8). Based on the self-administered online assessment, a cross-sectional study was conducted among professionals from different hospitals including MOH hospital, an academic institute, government hospital and private hospital. All the participants were currently working in those hospitals. Both genders were included in the study, where the number of females was 31, and the number of males was 64.

The study questionnaire included the initial details of the respondents including their nationality, age, education, gender and field of study. Afterwards, the questions about the average number of CT scan cases per duty that they have examined was asked. Furthermore, the investigations about the the most common examination performed during their duty was investigated as well as the common type of artifact they encounter while working; the common source of artifact they encountered in their department was investigated. The appropriate technique that was used to avoid or reduce artifact was also inquired. The questionnaire also asked about who was the responsible person for making decisions and managing quality control protocols.

Two types of data analysis methods (i.e. quantitative and qualitative) were performed. This study focused on the quantitative data analysis technique as the data were collected from survey. However, no data were collected in the form of students’ interviews; therefore, qualitative data analysis was not considered. In quantitative analysis, severe investigation was done to find the correlation between the variables selected in this study. The data were analysed using SPSS program version 20.0, and the results are represented in figures.

## Results

**Fig. 1:**
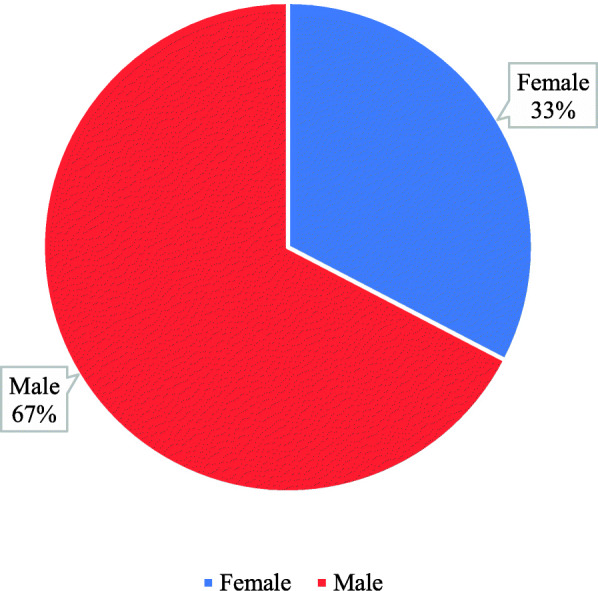
Gender distribution of the participants

Empirical assessment was performed considering the evaluation of the image’s artifacts in the computed tomography. More than half of the participants were males (i.e. 67%); on the other hand, 33% were females (Fig. 1).

**Fig. 2:**
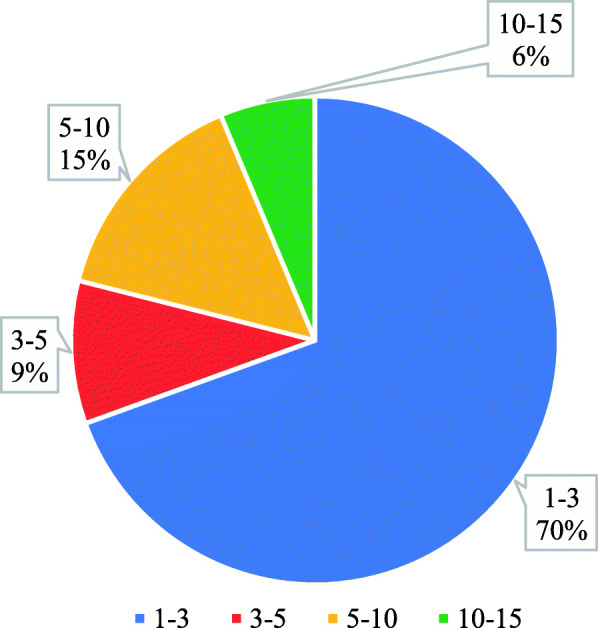
Practical experience of participants in CT scan department (year)

Practical experiences of the participants were assessed, and it was found that 70% of the participants had the experience of 1–3 years, 15% of the participants had the experience of 5–10 years, while 9% had the experience of 3–5 years (Fig. 2).

**Fig. 3:**
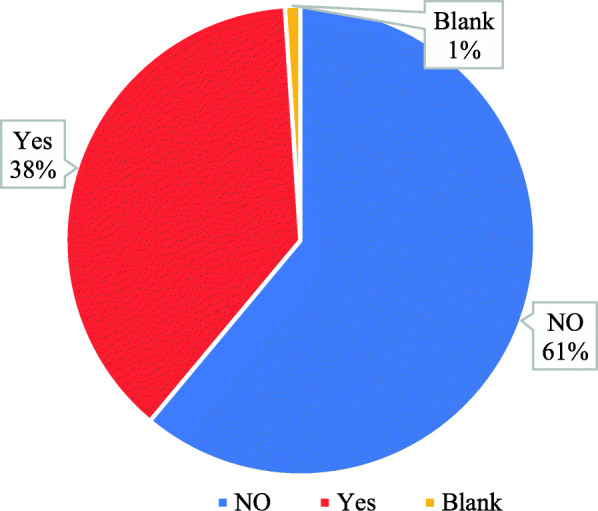
Subspeciality CT scan courses taken by the participants

The number of subspeciality CT scan courses taken by the participants was also inquired, and it showed that 38% of the participants had taken such courses, whereas 61% of the participants did not take any such courses; however, one participant left the space blank (Fig. 3).

**Fig. 4:**
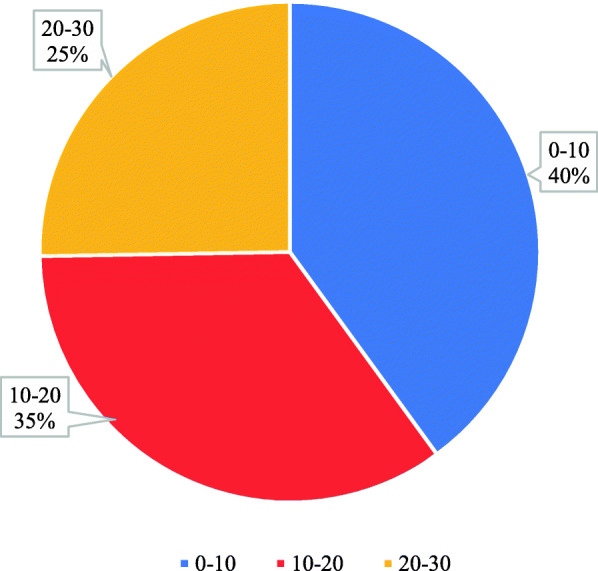
The average number of CT scan cases per duty witnessed by the participants

The participants were also asked about how many average number of CT scan cases they had witnessed per duty. It was found that 40% of participants answered 0–10 cases per day while 25% replied with 20–30 cases per day (Fig. 4).

**Fig. 5:**
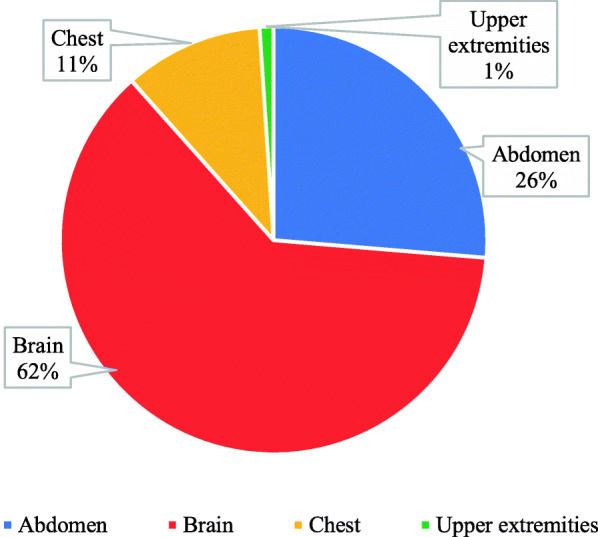
The most common examination performed during the duty

Participants were asked about the most common examination performed during their duty. It was found that 62% answered brain, 26% abdomen, and only 1% answered upper extremities (Fig. 5).

**Fig. 6:**
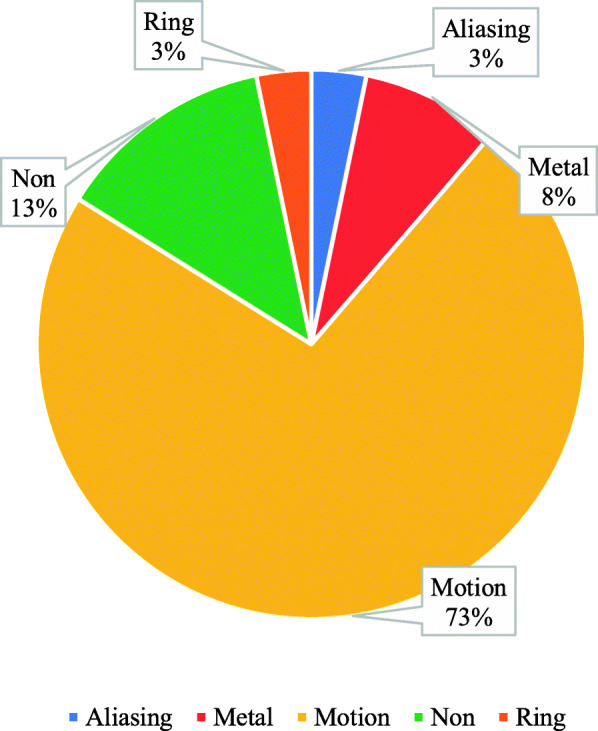
The common types of artifacts encountered while working

Participants were inquired about the common type of artifacts they encountered during their work. It was found that 73% of the participants answered with motion, while 3% answered with ring and aliasing respectively (Fig. 6).

**Fig. 7:**
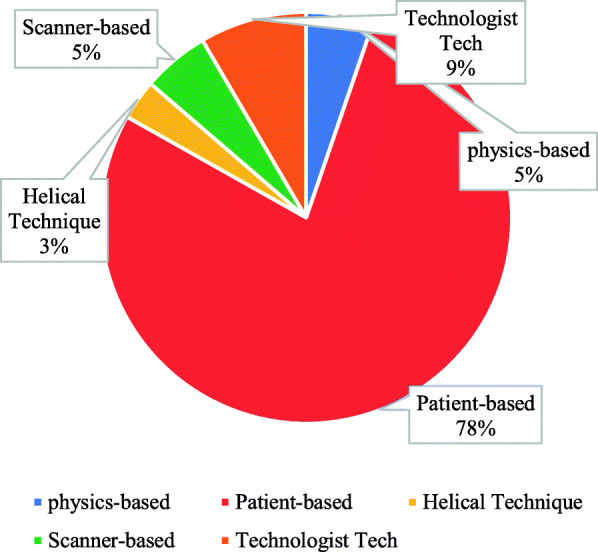
The common sources of artifacts encountered in the department

The common sources of artifacts participants encountered in their departments was also inquired, and it was found that 78% were patient-based sources, whereas 5% was physics-based (Fig. 7).

**Fig. 8:**
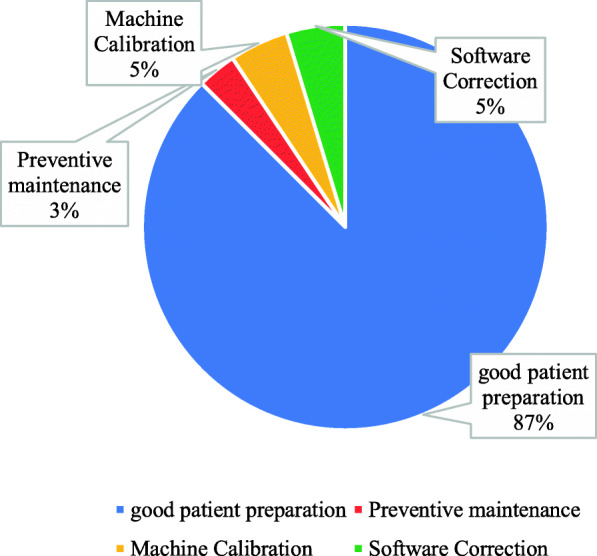
What is the appropriate technique used to avoid or reduce artifact

Participants were also inquired about the appropriate techniques utilized in order to avoid or reduce the artifact. It was shown that 87% of participants utilized good patient preparation, while 3% used preventive maintenance (Fig. 8).

**Fig. 9:**
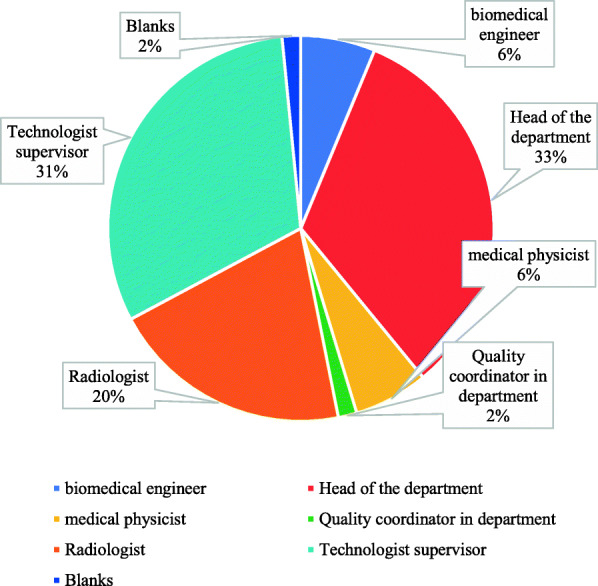
Who is the responsible person for making decisions and managing quality control protocols?

Participants were also asked about the responsible person for making decisions and managing quality control protocols. It was found that 33% answered the head of the department, whereas 6% answered biomedical engineer and medical physicist, respectively (Fig. 9).

## Discussion

Artifacts could degrade the image quality in the computed tomography, and sometimes making them diagnostically unusable; so, this study aimed to evaluate the common artifacts that effect on the image quality and the method of correction to improve image quality.

In this study, the common artifacts that appeared in the CT scan images was motion artifact (73%); however, this result was in contrast with Boas and Fleischmann [7]; they said, “Metal streak artifacts are very common, seen in 21% of scans in one sequence”,, because the motion artifact could be eliminated by rapid screening, and it depends on the CT technologist’s experience [7], while in this study, the majority of the CT technologists’ experience was between 1 and 3 years. On the other hand, the result was relevant to Yan et al. [8]; they reported, “In the procedure of CT scanning data collection, if in the fault the analyte have shifted, would lead to the projection data inconsistency so motion artifacts would be produced [8]. Normally, when scanning the patient, the patient’s moving, physiological movements such as the heart beating, breathing, and gastrointestinal peristalsis and the object entering or leaving the scanning plane. Severely injured patients or children frequently move during scanning, causing motion artifacts”.

The participants in this study were also asked about the common source of artifact in the CT departments; 78% of the participants answered the patient-based source, this is a logical result because the motion artifact was a common artifact in the CT departments; this finding was relevant to the study reported by Barrett [9]; he said, “the patient–based artifacts sources like metallic materials artifacts, which the presence of metal objects in the scan field could lead to severe streaking artifacts, and patient motion artifacts could cause mis-registration artifacts, which usually appeared as shading or streaking in the reconstructed image” [9].

Analysis has shown in this study that 62% of motion artifacts were presented in the brain CT because it was easier for the patient to move the head during CT exam; the second highest percentage was the abdomen by percentage 26% due to involuntary movement in the abdomen; these results were similar to Veikutis et al.’s [10]; they reported, “Analysis shown 29.9 % of artifacts presented in cerebral CT investigations, 24.3 % – thoracic, 16.6 % – spinal, 5.8 % – pelvic, and 2.0 % – abdominal [10]. The authors suggested that head is more prominent to motion; it’s easier for a patient to accidentally move head during CT scanning.”

Another study was reported by Kim et al. [11]; they said, “One of the major sources of image artifacts in computed tomography (CT) is patient motion, which created inconsistencies between acquired projections, leading to distortion and blurring when images are reconstructed [11]. These motion artifacts might lead to false diagnosis, or in extreme cases, render images uninterpretable.”

In the current study, the CT technologist considered that the patient preparation and communication are the appropriate techniques that they utilized in order to avoid or reduce the motion artifact; there is a research that has been taken by Boas and Fleischmann [7]; the authors mentioned that “Motion (patient, cardiac, respiratory or bowel) causes blurring and double images, as well as long-range streaks. The streaks occur between high-contrast edges and the x-ray tube position when the motion occurs [7]. Fast scanners reduce motion artifact because the patient has less time to move during the acquisition. This could be accomplished with faster gantry rotation or more x-ray sources. More detector rows allowed a greater volume to be imaged in a single gantry rotation, thus increasing the distance between step-off artifacts from motion on coronal or sagittal reformats. Rigid body motion artifacts (mainly a problem with head CT, could be reduced using special reconstruction techniques. Respiratory motion in cone-beam CT with slow gantry rotation could be estimated and corrected, thus reducing artifacts”. There is another research that has been taken by Zhou et al. [12], in which the author has mentioned certain procedures to reduce the metal artifact; they said, “The smart metal artifact reduction software” (SMAR) improves the quality of images and reduces artifacts to allow anatomic visualization of structures hidden underneath the artifacts by both subjective and objective measurements [12]. This results in improved diagnostic confidence in patients with a variety of implants.

In another study written by Wei et al. [13], they reported, “we found that compared to the 64-slice Discovery CT virtual monoenergetic images combined with MARS technique for image reconstruction, the 256-slice Revolution CT combined with Multi-material artifact reduction technique for image reconstruction is better to reduce metal artifacts and background standard deviation” [13].

There were some limitations of this study. Firstly, the number of the participants was only 95, as the questionnaire was distributed by social media, and there was no personal meeting with the CT technologist due to the current situation of the corona virus, thereby, larger samples are needed in further studies. Secondly, the results did not cover all the CT image artifacts due to the lack of available data; so, further studies should take into account all the CT scan image artifacts and the methods of correction.

## Conclusion

Artifacts decline the CT image quality and consequently obscure pathology, and it could originate from a range of sources, by using new designs in the scanner technology, careful positioning of patients during scanning, and optimal selection of scanner parameters (filter type, pitch, energy delivered), and most artifacts could be avoided. Some others could be minimized by solving the software development issues.

## Data Availability

The authors confirm that all data supporting the findings of the current study are available within the article. Also, all the datasets used and/or analysed during the current study are available from the corresponding author on a reasonable request.

## Abbreviations

CT: Computed tomography
R: Radiologist
SMAR: Smart metal artifact reduction
SPSS: Statistical Package for the Social Sciences
